# Based on Activation of p62-Keap1-Nrf2 Pathway, Hesperidin Protects Arsenic-Trioxide-Induced Cardiotoxicity in Mice

**DOI:** 10.3389/fphar.2021.758670

**Published:** 2021-10-13

**Authors:** Yuxin Jia, Jing Li, Panpan Liu, Mingdong Si, Yanyu Jin, Hongfang Wang, Donglai Ma, Li Chu

**Affiliations:** ^1^ School of Pharmacy, Hebei University of Chinese Medicine, Shijiazhuang, China; ^2^ Hebei Key Laboratory of Integrative Medicine on Liver-Kidney Patterns, Hebei University of Chinese Medicine, Shijiazhuang, China; ^3^ Hebei Higher Education Institute Applied Technology Research Center on TCM Formula Preparation, Shijiazhuang, China

**Keywords:** arsenic trioxide, cardiotoxicity, hesperidin, oxidative stress, p62-Keap1-Nrf2 pathway

## Abstract

**Background:** Hesperidin (HES) is a flavonoid glycoside found in the tangerine peel and has antioxidant properties. Arsenic trioxide (ATO) is an anti-tumour drug; however, its serious cardiotoxicity limits its clinical application. In addition, the protection of HES against ATO-induced cardiotoxicity has not been explored.

**Objective:** The study aims to investigate and identify the underlying effect and mechanism of HES on ATO-induced cardiotoxicity.

**Methods:** Fifty mice were randomly assigned to five groups. Mice were orally given HES:100 or 300 mg/kg/day concurrently and given ATO intraperitoneal injections: 7.5 mg/kg/day for 1 week. Blood and heart tissues were collected for examination. Evaluated in serum was the levels of creatine kinase (CK), lactate dehydrogenase (LDH) and cardiac troponin I (cTnI). In addition, evaluated in heart tissues were the levels of reactive oxygen species (ROS), superoxide dismutase (SOD), malondialdehyde (MDA), glutathione (GSH), catalase (CAT), tumour necrosis factor-α (TNF-α), interleukin-6 (IL-6), B-cell lymphoma-2 (Bcl-2), Bcl-2-associated X protein (Bax), Caspase-3, cleaved-Caspase-3, p62, Kelch-like ECH-associated protein 1 (Keap1), and nuclear factor erythroid 2-related factor 2 (Nrf2). The heart tissues were also examined for histopathology and mitochondrial ultrastructure.

**Results:** Compared with the ATO group, the HES treatment groups reduced the levels of CK, LDH, cTnI, ROS, MDA, TNF-α, IL-6, Bax, Caspase-3, cleaved-Caspase-3 and Keap1 and enhanced the levels of SOD, GSH, CAT, Bcl-2, p62 and Nrf2.

**Conclusions:** The results demonstrate that HES protects against ATO-induced cardiotoxicity, through inhibiting oxidative stress, and subsequent inflammation and apoptosis. The underlying results are closely related to the regulation of the p62-Keap1-Nrf2 signalling pathway.

## Introduction

Acute promyelocytic leukaemia (APL) is a distinct type of acute myeloid leukaemia, which has a “rapid downhill course” and is characterised by a severe bleeding tendency (Hillestad, 1957). Arsenic trioxide (ATO) is an active ingredient in traditional Chinese medicine and has been recognised as the best drug for APL (Kayser et al., 2018). Unfortunately, during the APL treatment, the application of high doses of ATO is severely toxic against organs, greatly limits its use. The heart is one of the organs that is seriously affected by ATO poisoning. The main cardiotoxicity manifestations are QT prolongation, torsades de pointes, and sudden cardiac death (Barbey and Soignet, 2001; Unnikrishnan et al., 2001; Westervelt et al., 2001). These events may increase the prevalence of cardiovascular disease in APL patients who have been offered ATO therapy. Due to these limitations, many patients are unwilling to undergo ATO therapy. Therefore, it is important to explore the cardiac toxicity associated with ATO therapy and seek for appropriate attenuating drugs to improve its clinical application.

Recent studies have demonstrated that oxidative stress injury, inflammation and apoptosis of myocardial cells are potential mechanisms associated with ATO-induced cardiotoxicity, in which oxidative stress is the main cause (Chang et al., 2007; Das et al., 2010; Ye et al., 2010; Birari et al., 2020; Xue et al., 2020). Reactive oxygen species (ROS) are produced through a series of extracellular and intracellular activities, considered as a novel signalling mediator involved in cell growth, differentiation, progression and death (Sena and Chandel, 2012; Zhang et al., 2013a). Elevated intracellular ROS levels provoke lipid, protein and DNA damage, attributed to oxidative stress (Schieber and Chandel, 2014). In addition, oxidative stress activates transcription factors that release pro-inflammatory cytokines and trigger an inflammatory response (Lee and Yang, 2012; Turillazzi et al., 2016). Oxidative stress can also cause oxidative damage to mitochondria, thereby increasing the release of pro-apoptotic proteins and leading to apoptosis (Kumar et al., 2014; Zhao et al., 2017). Other studies identified the Kelch-like ECH-associated protein 1 (Keap1)-nuclear factor erythroid 2-related factor 2 (Nrf2) pathway as the primary cellular defence mechanism against oxidative stress (Canning et al., 2015; Chen and Maltagliati, 2018; Cai et al., 2021; Yu et al., 2021; Zhao et al., 2021). In addition, p62 is a crucial factor in the regulation of the Keap1-Nrf2 pathway (Kansanen et al., 2013; Bartolini et al., 2018).

**FIGURE 1:**
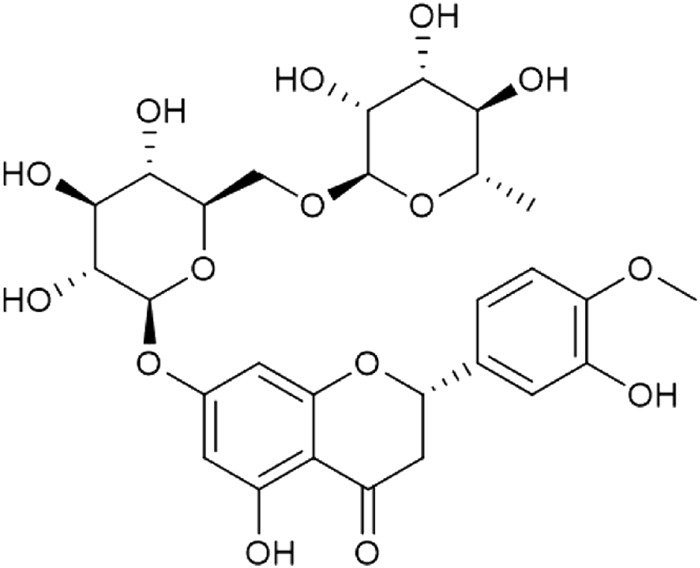
Chemical structure of HES.

Hesperidin (HES; Figure 1) is a flavonoid glycoside derived from the tangerine peel, has anti-inflammatory, antioxidant and anti-apoptosis properties (Kumar et al., 2013; Li and Schluesener, 2017; Afzal et al., 2020). Some studies confirm that HES protects against arrhythmia and heart failure caused by myocardial ischemia/reperfusion (He et al., 2017) and anti-cancer drugs such as doxorubicin (Hozayen and Seif, 2011) and cisplatin (Oguzturk et al., 2016), demonstrating that HES can be used as a good cardiac protectant. Nevertheless, no research has demonstrated that HES can be used as a protective agent against ATO-induced cardiotoxicity or its underlying mechanism.

This present study establishes a cardiotoxicity model by administering ATO to Kunming mice, aiming to investigate the protection of HES against ATO cardiotoxicity from oxidative stress, inflammation and apoptosis. The underlying mechanism is closely related to the p62-Keap1-Nrf2 signalling pathway regulation. Our study could improve the safety of ATO and confirm the HES cardioprotective effects.

## Materials and Methods

### Drugs

ATO (Beijing Shuanglu Property Management Co., Ltd., Beijing, China) was dissolved in normal saline (NS) to reach an appropriate concentration before administration (Sun et al., 2021). HES (Tokyo Chemical Industry Co., Ltd, Tokyo, Japan) was prepared as a suspension, dissolving it in 0.5% sodium carboxymethyl cellulose (0.5% CMC-Na) and shaking vigorously before use (Li et al., 2021).

### Animals

Fifty male Kunming mice (20 ± 2 g, 4-week-old), obtained from Hebei Medical University (Certificate No. SCXK (Hebei) 2018-004), were kept in the Research Centre of Hebei University of Chinese Medicine. All mice were kept in a standard environment with a constant temperature of 23 ± 2°C, relative humidity of 45-60% and a 12 h light-dark cycle. All mice were fed adaptively for 1 week, allowing them to eat and drink freely. The experimental design was approved by the Ethics Committee for Animal Experiments, Hebei University of Chinese Medicine (approval number: DWLL 2021056; approval date: March 2, 2021).

### Experimental Design

The 50 mice were randomly grouped into 5, with 10 mice in each group. During the experiment, mice in each group were given 90 g of normal feed and 200 ml of purified water every morning, and the remaining quantity of food and water was recorded the next morning to compare the effects of drugs on diet and water intake. The body weights of mice in each group were recorded before the first administration and after the last administration. After a 7-days adaptation period, all mice entered the administration phase. The administration protocol was as follows:

Control group (CONT): 0.5% CMC-Na (isovolumetric of HES groups, oral) + NS (isovolumetric of ATO group, i.p.)

ATO group (ATO): 0.5% CMC-Na (isovolumetric of HES groups, oral) + ATO 7.5 mg/kg/day (i.p.)

Low-HES + ATO group (HES_L_): HES 100 mg/kg/day (oral) + ATO 7.5 mg/kg/day (i.p.)

High-HES + ATO group (HES_H_): HES 300 mg/kg/day (oral) + ATO 7.5 mg/kg/day (i.p.)

HES group (HES): HES 300 mg/kg/day (oral) + NS (isovolumetric of ATO group, i.p.)

The dose selection for ATO and HES followed Jin, et al. (Jin et al., 2020) and Erdinç, et al. (Turk et al., 2019), respectively. HES was administered orally 6 h once daily for 7 days before ATO intraperitoneal treatment. Adequate diet and drinking water were offered during administration. After the last administration, the mice were left to fast for 24 h, and then samples were quickly collected.

### Sample Collection

Mice were anaesthetized with sodium pentobarbital (50 mg/kg, i.p.), and blood samples were drawn from the retro-orbital venous plexus and centrifuged at 3,500 rpm for 10 min. The supernatant was extracted and stored at −20°C for later use. Afterwards, the chests of the mice were opened, and their hearts were quickly removed. The hearts in each group were cut and fixed in 4% paraformaldehyde or 2.5% glutaraldehyde phosphate buffer. The rest of the hearts were frozen in liquid nitrogen or stored in a refrigerator at −80°C for later index detection.

### Hematoxylin-Eosin Staining

Heart tissue, fixed in 4% paraformaldehyde for 48 h, was moved out for dehydration and embedded in paraffin to prepare for sectioning. Then, the myocardial sections (4 μm) were stained with H&E. Finally, the pathological changes of myocardial tissue were observed under an optical microscope (Leica DM4000B, Solms, Germany). The degree of myocardial injury was quantified by Image-Pro Plus 6.0 software.

### Serum Biochemical Analysis

We obtained the blood supernatant through centrifugation and was used to determine the levels of related cardiac biomarkers in serum according to the guidelines of the commercially available kits. Total serum was marked as creatine kinase (CK) (Jian Cheng Biological Engineering Institute, Nanjing, China; Catalog number: A032-1-1), lactate dehydrogenase (LDH) (Jian Cheng Biological Engineering Institute, Nanjing, China; Catalog number: A020-1-2) and cardiac troponin I (cTnI) (Sigma Co., Ltd, MO, United States; Catalog number: SEKM-0153). Changes in these indicators correlate closely with alterations in heart function.

### ROS Expression

Dihydroethidium (DHE) oxidation is a classic method to determine ROS content. The cardiac tissues of each group were incubated with DHE (Sigma. Co., Ltd, MO, United States; Catalog number: D7008) at 37°C for 30 min in the dark, washed with Phosphate Buffered Saline (PBS) 3 times, incubated with DAPI staining solution (Servicebio Technology Co., Ltd, Wuhan, China; Catalog number: G1012) at room temperature for 10 min without light and washed again with PBS 3 times. ROS oxidized DHE is incorporated into chromosomal DNA, making the nucleus show red fluorescence. The content of ROS was assessed through the production of red fluorescence under a fluorescence microscope (Leica DM4000B, Solms, Germany), and the ROS intensity in the images was digitized by Image-Pro Plus 6.0.

### Biochemical Index Analysis of Heart Tissue

A certain amount of tissue was weighed to produce 10% heart homogenate with 9 times as much NS. After centrifugation at 2,500 rpm for 10 min, the supernatants were retained for detection. The levels of superoxide dismutase (SOD, Catalog number: A001-3-1), malondialdehyde (MDA, Catalog number: A003-2-2), glutathione (GSH, Catalog number: A006-2-1), and catalase (CAT, Catalog number: A007-1-1) in heart tissue were measured according to the instructions of the appropriate kits (Jian Cheng Biological Engineering Institute, Nanjing, China).

### Inflammatory Cytokine Analysis

Nine times the volume of PBS (pH 7.4) were added to the heart tissue to prepare a 10% buffered homogenate, and the supernatants of the cardiac homogenate samples were collected after centrifugation at 3,000 rpm for 10 min. Detected based on the ELISA kits procedures were the inflammatory cytokines tumor necrosis factor-α (TNF-α) (Thermo Fisher Scientific Inc., Massachusetts, America; Catalog number: 88–7324) and interleukin-6 (IL-6) (Multi Sciences, Biotech, Co., Ltd, Hangzhou, China; Catalog number: EK206/3-01), in the supernatant of tissue homogenate.

### Mitochondrial Ultrastructure Analysis

Approximately 1 mm^3^ of heart tissue was removed, immediately immersed in 2.5% glutaraldehyde phosphate buffer, fix at 4°C for 3°h, then fixed again with 1% osmic acid in 0.1 mol/L phosphate buffer (pH 7.4) for 2 h at room temperature. The fixed tissues were dehydrated, soaked, embedded and polymerized to make ultrathin sections. The ultrastructure of mitochondria was observed and photographed under a transmission electron microscopy (TEM) (HT7800, Hitachi, Japan) at 80.0 kV.

### Apoptosis and Pathway-Related Protein Analysis

RIPA lysates (Servicebio Technology Co., Ltd, Wuhan, China; Catalog number: G2002) and phenylmethylsulfonyl fluoride were added to PBS-cleaned heart tissues, afterwards crushed into homogenates. The myocardial tissue homogenate was centrifuged at 12,000 rpm for 30 min at 4°C, and the supernatant was reserved for the next experiments.

The proteins were separated by 10% sodium dodecyl sulfate-polyacrylamide gel electrophoresis and transferred onto polyvinylidene fluoride membranes. After blocking with 5% skimmed milk in TBST for 2 h at room temperature, the primary antibodies were incubated on the membrane overnight at 4°C. After incubating with the primary antibody, the membrane was washed with TBST 3 times for each 10 min. Then, we incubated the secondary antibody at room temperature for 2 h and washed the membrane as described above. After scanning the film, proteins were visualized and quantified using Image-Pro Plus 6.0 software.

The primary antibodies used in this study were anti-Bcl-2-associated X protein (Bax) (Abways Biotechnology Co., Ltd., Shanghai, China; Catalog number: CY5059), anti-B-cell lymphoma-2 (Bcl-2) (Abways Biotechnology Co., Ltd., Shanghai, China; Catalog number: CY5032), anti-Caspase-3 (Abways Biotechnology Co., Ltd., Shanghai, China; Catalog number: CY5051), anti-cleaved-Caspase-3 (Cell Signaling Technology Inc., Danvers, MA, United States; Catalog number: CST9664), anti-p62 (Servicebio Technology Co., Ltd., Wuhan, China; Catalog number: GB11531), anti-Keap1 (Proteintech Group, Inc., Wuhan, China; Catalog number: 10503-2-AP), and anti-Nrf2 (Proteintech Group, Inc., Wuhan, China; Catalog number: 16396-1-AP), which were diluted at 1:1,000. Anti-β-actin (ABclonal Technology Co., Ltd., Wuhan, China; Catalog number: AC026) and anti-Lamin B1 (Proteintech Group, Inc., Wuhan, China; Catalog number: 66095-1-Ig) were used as a standard to normalize the total and nuclear protein expressions, respectively. The secondary antibodies comprise horseradish peroxidase-conjugated goat anti-rabbit IgG (Bioeasy Technology Co., Ltd., Beijing, China, Catalog number: BE0101), diluted in 1:10,000.

### Data Analysis

IBM SPSS Statistics software version 21.0 was used for the analysis, and experimental data of each group were presented as the mean ± standard deviation (SD). One-way analysis of variance (ANOVA) and Tukey’s tests were used to compare groups and carry out the pair comparisons between groups. *p*-value < 0.05 was used as the criterion for statistically significance.

## Results

### HES Toxicity Analysis

**FIGURE 2:**
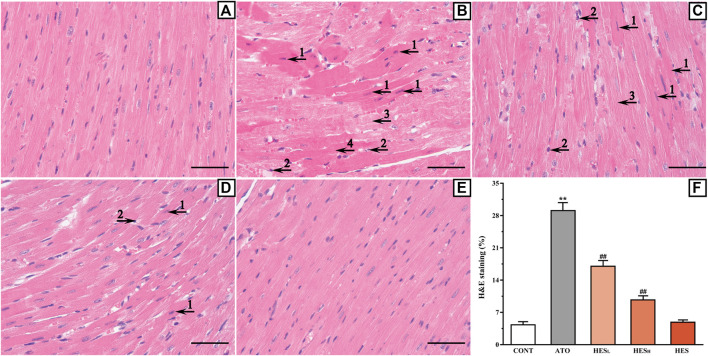
Histopathological changes of heart between ATO group and HES treatment groups (scale bar = 50 μm, magnification: ×400). The results of H&E staining shown in the A to E represent the CONT, ATO, HES_L_, HES_H_ and HES groups, respectively. Arrow 1 points to apoptotic cells; arrow 2 points to inflammatory cells; arrow 3 points to myocardial oedema cells, and arrow 4 points to myocardial necrosis.

**FIGURE 3:**
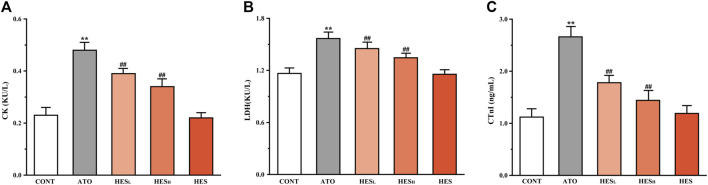
Changes in CK **(A)**, LDH **(B)** and cTnI **(C)** expression are shown between ATO group and HES treatment groups. Values are expressed as mean ± SD (*n* = 6). Compared with CONT group (**p < 0.01), compared with ATO group (^#^ *p* < 0.05, ^##^ *p* < 0.01).

**FIGURE 4:**
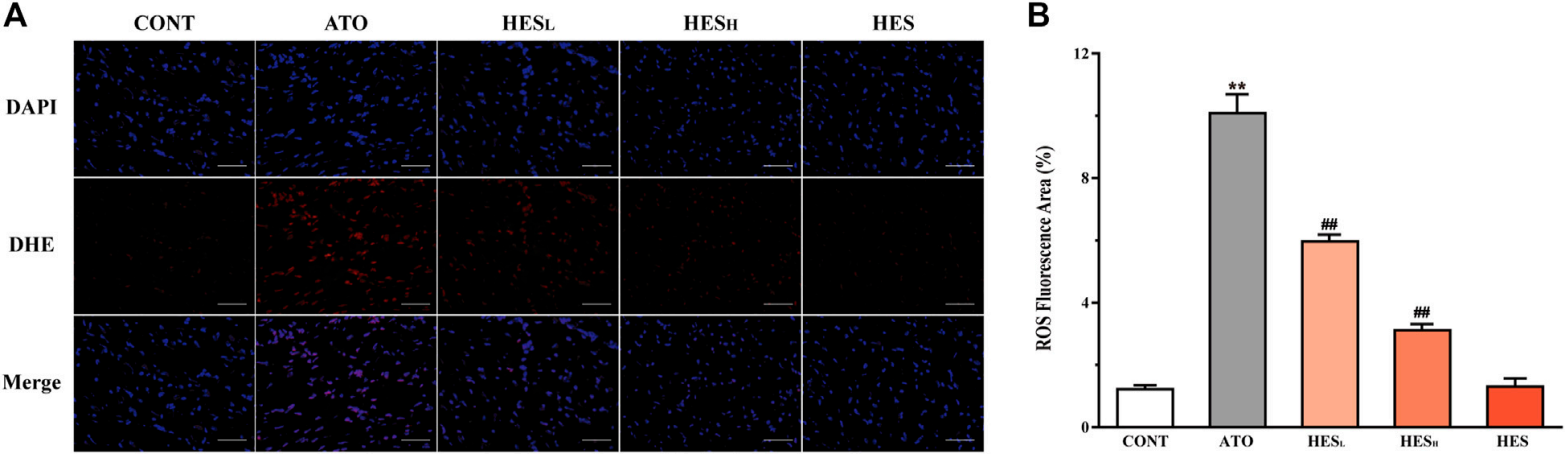
Changes in ROS expression between ATO group and HES treatment groups. **(A)** Detected ROS expression in myocardial tissue with DHE probe, scale bar = 50 μm. **(B)** values are expressed as mean ± SD. ^**^ *p* < 0.01, compared with CONT group, ^##^ *p* < 0.01, compared with ATO group.

**FIGURE 5:**
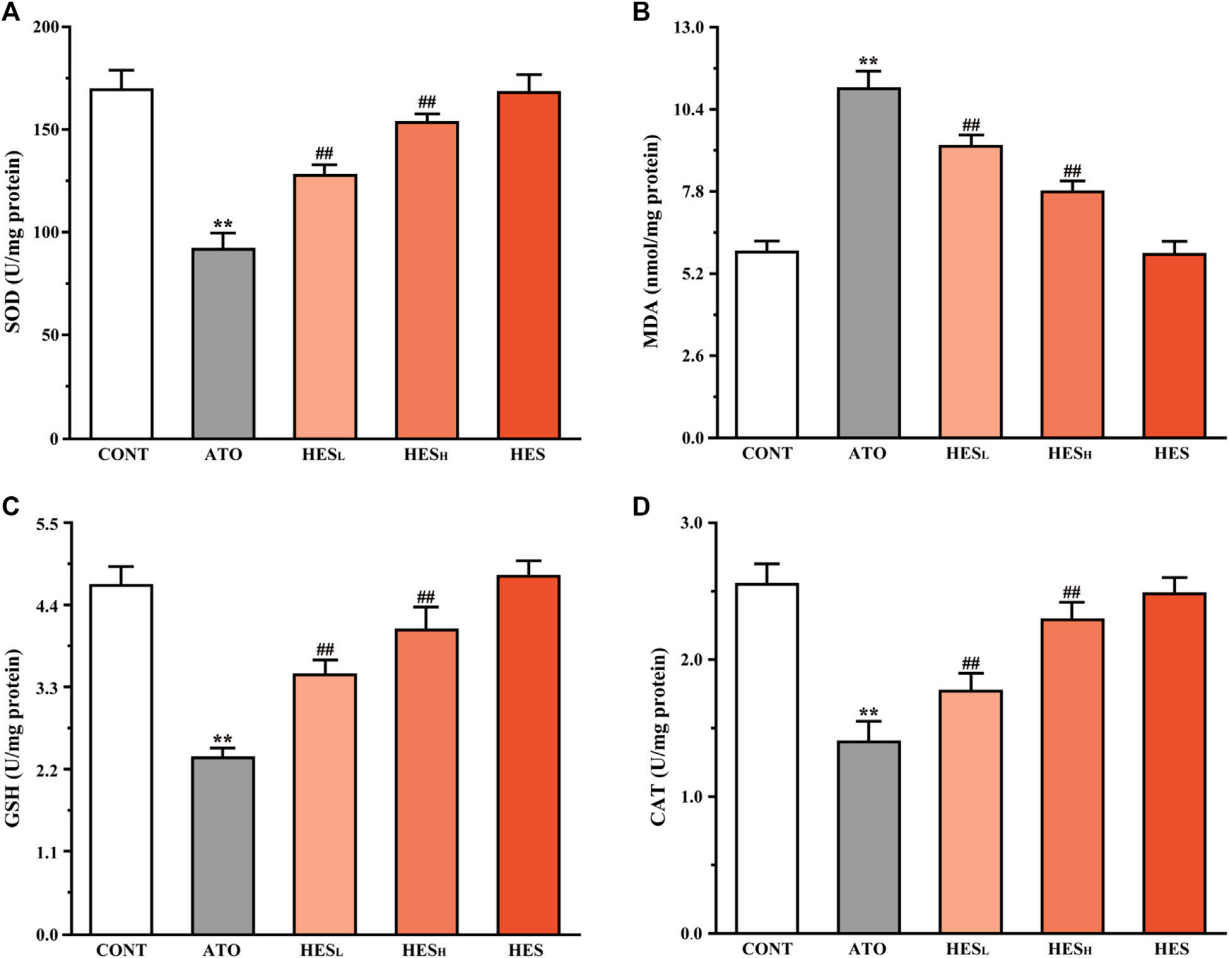
Changes in SOD **(A)**, MDA **(B)**, GSH **(C)**, and CAT **(D)** expression between ATO group and HES groups. Results are expressed as mean ± SD (*n* = 6). **p < 0.01, compared to CONT group, ^##^ *p* < 0.01, compared to ATO group.

**FIGURE 6:**
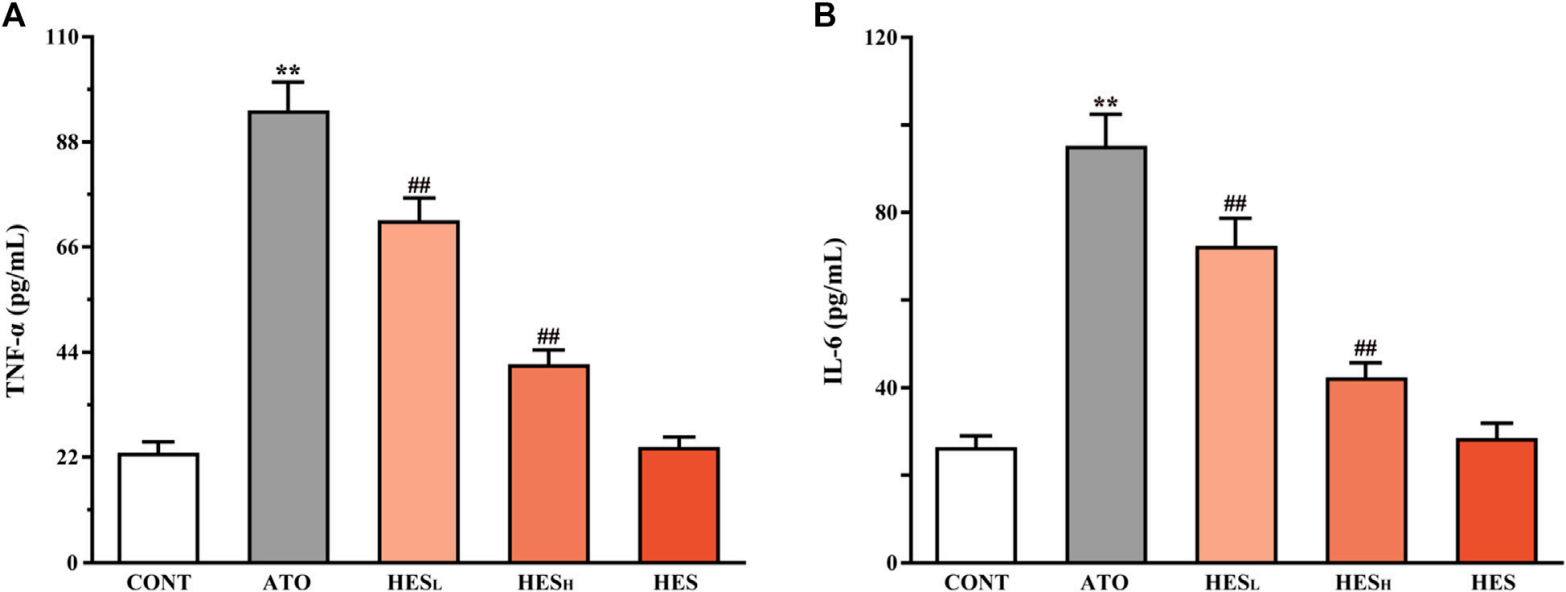
Changes in TNF-α **(A)** and IL-6 **(B)** between the ATO group and HES treatment groups. Results are expressed as mean ± SD (*n* = 6). **p < 0.01, compared to CONT group, ^##^ *p* < 0.01, compared to ATO group.

**FIGURE 7:**
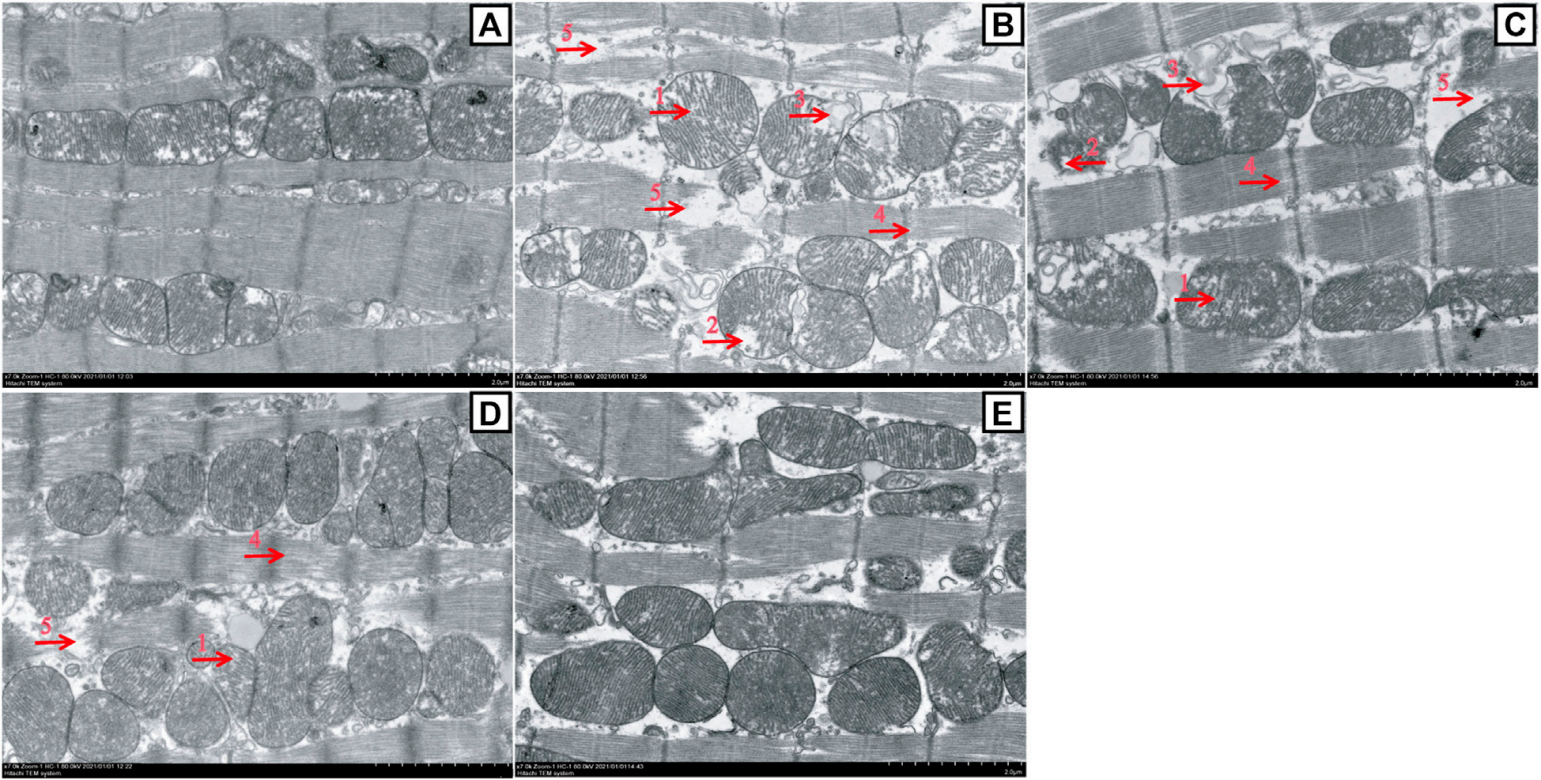
Changes in myocardial mitochondrion ultrastructure between the ATO group and HES treatment groups (scale bar = 2.0 μm, magnification: 7000 ×). A to E represent the CONT, ATO, HES_L_, HES_H_, and HES groups, respectively. TEM images show the ultrastructure changes of myocardial mitochondria: the mitochondria were swollen, and the cristal space was enlarged (arrow 1); the crest became short or dissolved and disappeared (arrow 2); the cristae disappeared, and the mitochondria were vesicular (arrow 3); the space between myofilaments was enlarged (arrow 4); and myofilaments broke, dissolved and disappeared (arrow 5).

**FIGURE 8:**
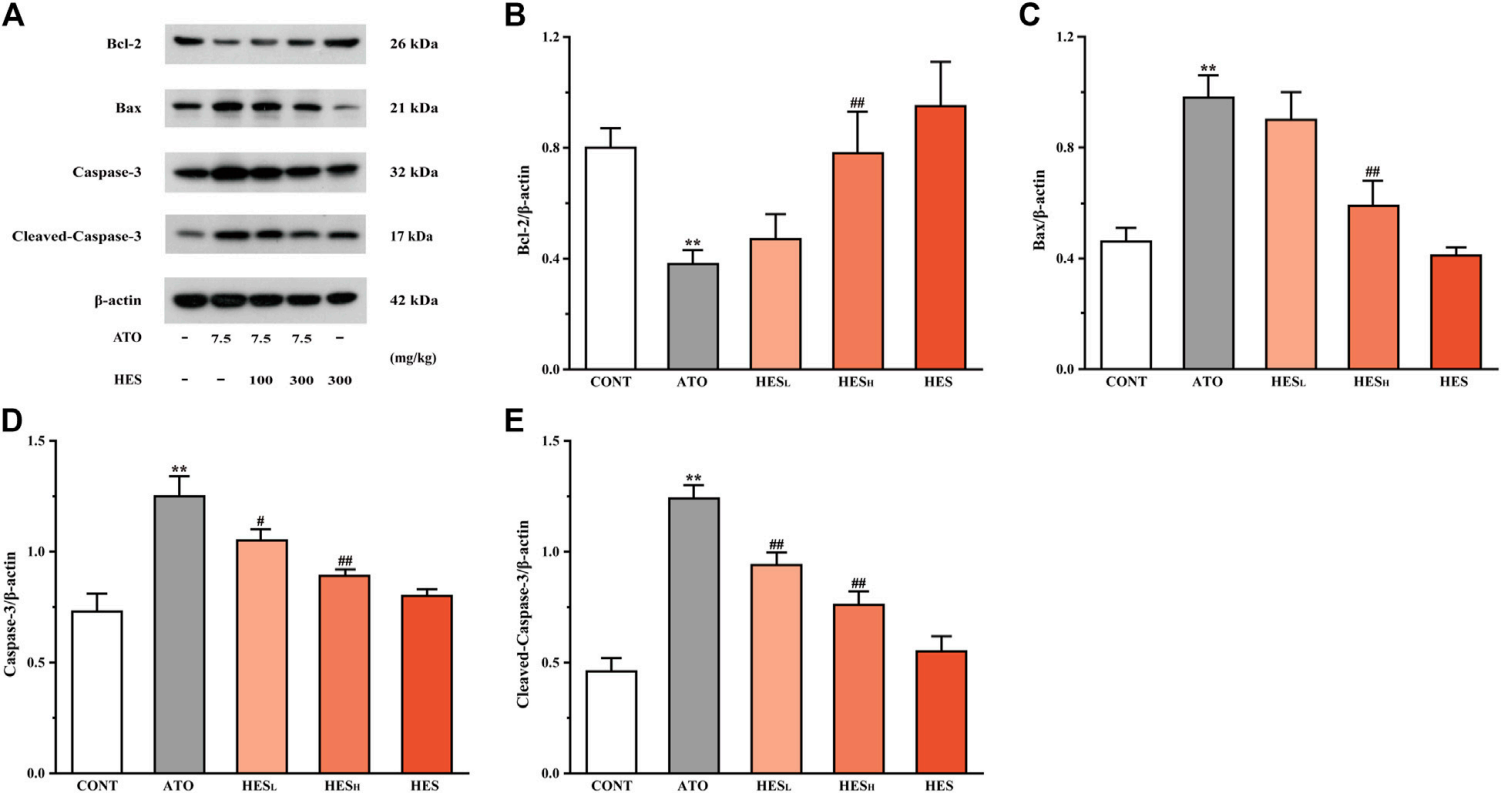
Changes in Bcl-2, Bax, Caspase-3 and cleaved-Caspase-3 protein expressions between ATO group and HES treatment groups. Results are expressed as mean ± SD (*n* = 3). **p < 0.01, compared to CONT group, ^##^ *p* < 0.01, compared to ATO group.

Compared with mice in the CONT group, mice in the HES group did not exhibit any abnormalities or deaths during the experiment (Table 1). The test results of serum histopathology (Figure 2), biochemical indexes (Figure 3), oxidative stress indexes (Figures 4,5), inflammatory factors (Figure 6), and apoptosis (Figure 7,8) did not show any statistical differences between the HES group and CONT group. These comparisons suggest a HES safety effect.

**TABLE 1:** Summary of general observations in mice.

Traditional recording	CONT	ATO	HES_L_	HES_H_	HES
Mean food consumption (g/mouse/day)	5.77 ± 0.25	4.91 ± 0.20^**^	5.26 ± 0.28	5.59 ± 0.28^##^	5.92 ± 0.32
Mean water consumption (mL/mouse/day)	7.05 ± 0.28	5.40 ± 0.62^**^	6.46 ± 0.20^##^	6.74 ± 0.28^##^	7.12 ± 0.29
Initial body weight (g)	27.93 ± 1.61	27.17 ± 1.97	27.99 ± 1.70	27.89 ± 1.50	27.95 ± 1.76
Final body weight (g)	32.95 ± 1.57	29.25 ± 1.66^**^	30.95 ± 1.88	32.26 ± 1.75^##^	33.19 ± 1.95
Mortality (ratio %)	0.00	0.00	0.00	0.00	0.00

Note: Data are presented as mean ± SD (*n* = 10) or as a ratio. Compared to the CONT group (^**^ *p* < 0.01), compared to the ATO group (^#^ *p* < 0.05, ^##^ *p* < 0.01).

### Effects of HES on a Diet, Water Intake and Body Weight in Mice

During treatment, general observations showed that the average daily intake of water and diet in the ATO group was significantly different from that of the CONT group (*p* < 0.01; Table 1). At the end of the experiment, the average body weight of mice in the ATO group significantly reduced compared with the CONT, HES_L_, and HES_H_ groups (*p* < 0.05 or *p* < 0.01). Moreover, none of the mice died during the experiment.

### Effects of HES on ATO-Induced Histopathological Changes

As shown in Figure 2, H&E staining confirmed that a variety of pathological changes occurred in the ATO group (Figure 2B) compared with the CONT group (Figure 2A). Tissue lesions in the HES_L_ and HES_H_ groups (Figures 2C,D) decreased as the dose increased compared with the ATO group (*p* < 0.01). These changes were manifested based on the presence of apoptotic cells, the appearance of inflammatory cells, the enlargement of the fibrous space in the myocardium (cardiomyocyte oedema) and the occurrence of myocardial necrosis.

### Effects of HES on ATO-Induced Changes in Myocardial Enzyme

In Figure 3, the results of the serum biochemical parameters showed that ATO administration significantly increased the levels of CK (Figure 3A), LDH (Figure 3B) and cTnI (Figure 3C) compared to the CONT group (*p* < 0.01). In the HES_L_ and HES_H_ groups, downward trends were observed in the levels of the serum biomarkers of cardiac injury, in contrast to the ATO group (*p* < 0.05 or *p* < 0.01).

### Effects of HES on ATO-Induced Changes in ROS Expression

In Figure 4, fluorescence results show that the ROS fluorescence intensity of the ATO group was significantly enhanced compared to the CONT group (*p* < 0.01). The HES_L_ and HES_H_ treatments effectively restrained ROS overexpression induced by the ATO (*p* < 0.01).

### Effects of HES on ATO-Induced Changes in Oxidation and Antioxidant Indices

In Figure 5, the results of the heart tissue biochemistry showed that the ATO group significantly increased in MDA content (*p* < 0.01; Figure 5B) and decreased in SOD (Figure 5A), GSH (Figure 5C), and CAT (Figure 5D) levels compared to the CONT group (*p* < 0.01). However, HES_L_ and HES_H_ treatment increased the levels of SOD, GSH, and CAT (*p* < 0.01), while decreasing the content of MDA (*p* < 0.01).

### Effects of HES on ATO-Induced Changes in TNF-α and IL-6 Release

In Figure 6, results from ELISA showed that ATO induced higher expression of inflammatory factors TNF-α and IL-6 than in the CONT group (*p* < 0.01). However, the levels of TNF-α and IL-6 in heart tissues of the HES_L_ and HES_H_ groups were lower than the levels in the ATO group (*p* < 0.01).

### Effects of HES on ATO-Induced Changes in Myocardial Mitochondrion Ultrastructure

In Figure 7, the TEM images showed that the myocardial mitochondria had a normal morphological structure in the CONT group (Figure 7A). However, almost all the myocardial mitochondria in the ATO group were damaged (Figure 7B), including the widening of the cristal space (mitochondrial swelling), the shortening, breaking or disappearance of the crest (mitochondria appear vesicular), the enlargement of filament space in myofibrils (myocardial cells had slight oedema), and the fracture, dissolution and disappearance of some myofilaments. HES_L_ and HES_H_ treatment prevented the development of ATO-induced mitochondrial damage (Figures 7C,D).

### Effects of HES on ATO-Induced Changes in Apoptosis

In Figure 8, the results of the western blot of apoptosis-related proteins showed that the Bcl-2 expression was down-regulated while the expressions of Bax, Caspase-3 and cleaved-Caspase-3 were up-regulated in the ATO group when compared to the CONT group (*p* < 0.01). However, compared with the ATO group, mice in the HES_L_ and HES_H_ groups increased anti-apoptotic protein (Bcl-2) expression, while the expression of pro-apoptotic proteins was restricted (Bax, Caspase-3 and cleaved-Caspase-3) (*p* < 0.05 or *p* < 0.01).

### Effects of HES on ATO-Induced Changes in p62, Keap1 and Nrf2 Expression

**FIGURE 9:**
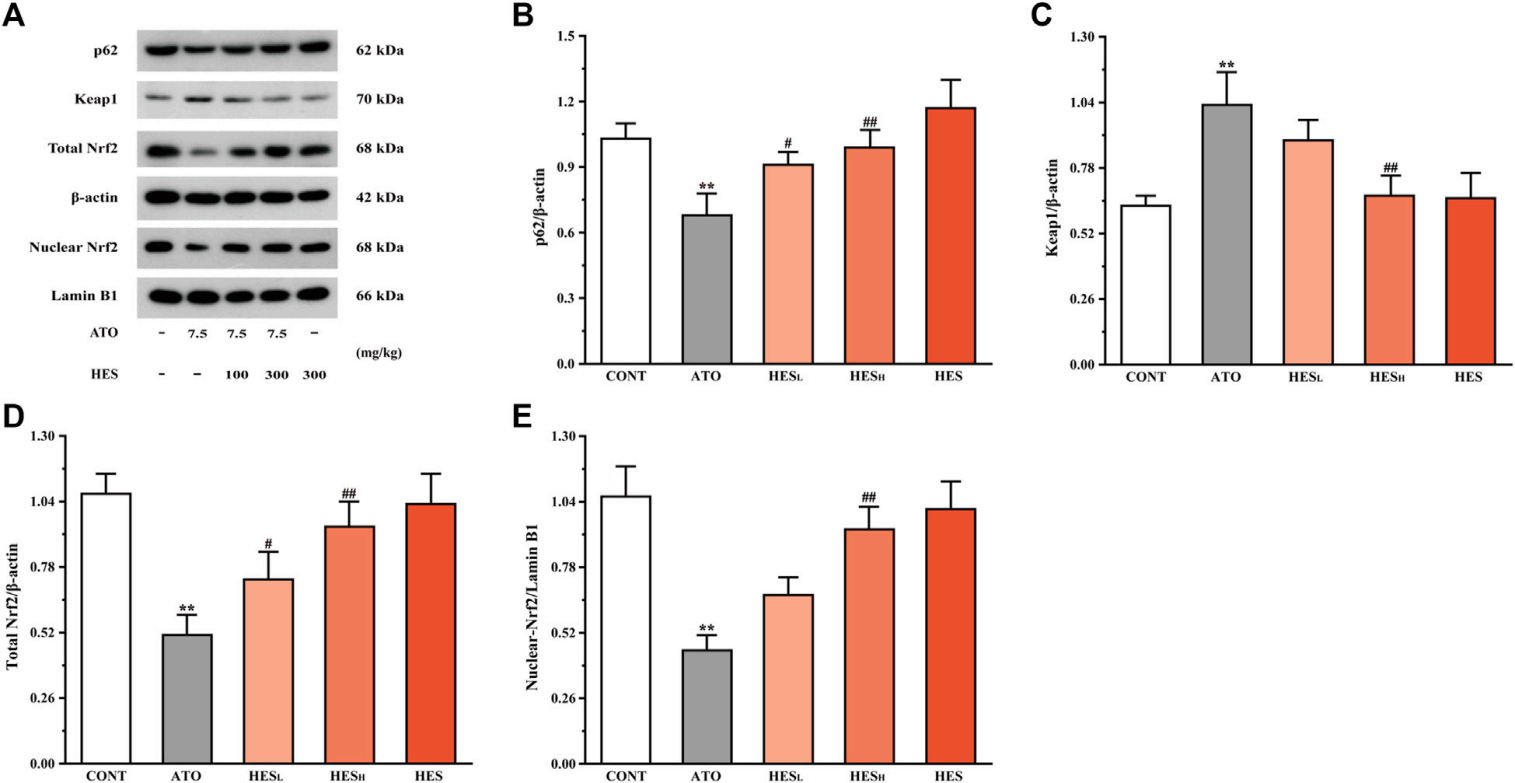
Changes in p62, Keap1, and Nrf2 protein expressions between ATO group and HES groups. Results are expressed as mean ± SD (*n* = 3). **p < 0.01, compared to CONT group, ^#^ *p* < 0.05 and ^##^ *p* < 0.01, compared to ATO group.

In Figure 9, the western blot results of pathway-related proteins show a dramatic increase in the Keap1 level and an obvious decrease in the levels of p62 and Nrf2 in the ATO group when compared to the CONT group (*p* < 0.01). Conversely, after HES_L_ and HES_H_ treatment, the expression of p62 and Nrf2 proteins were up-regulated, and the expressions of Keap1 were down-regulated (*p* < 0.05 or *p* < 0.01).

## Discussion

ATO is a broad-spectrum drug plays a critical role in the anti-tumour development and offers remarkable efficiency in the treatment of patients with APL. However, the ATO is cardiotoxic. As a result, patients are prevented from accepting this high-efficient therapy (Antman, 2001; Vineetha and Raghu, 2019). Finding an appropriate antidote is crucial for the treatment of ATO-related cardiotoxicity to improve the ATO positive clinical effect.

In the present study, mice in the ATO group experience significant heart damage, leading to reductions in weight, food intake, and water consumption (Table 1). This group also demonstrates different pathological changes, which include apoptotic cells, inflammatory cells, and myocardial necrosis in myocardial tissue (Figure 2). Myocardium pathological changes are some of the crucial indicators to assess myocardial injury (Fu et al., 2015). However, HES treatment improved weight, food intake and water consumption of the mice as well as alleviating histopathological damage. This suggests that HES may have a protective effect against ATO-induced cardiotoxicity.

The CK and LDH are myocardial markers, and the level of myocardial enzymes in serum reflects the degree of myocardial cell damage (Liang et al., 2020; Zheng et al., 2021). In addition, cTnI is a well-known specific biomarker choice for the detection of cardiac dysfunction. Moreover, myocardial injury is triggered by excessive cTnI release (Amran et al., 2015). In the current study, the ATO group presented a significant increase in CK, LDH and cTnI levels compared with the CONT group, which was consistent with previous results (Amran et al., 2015; Liang et al., 2020; Zheng et al., 2021). However, the leak of myocardial markers significantly reduced in the HES_L_ and HES_H_ groups, indicating that HES played a cardioprotective role by reducing the release of the myocardial enzymes (Figure 3).

Some studies confirmed that ROS were the major source of ATO-induced cardiotoxicity (Aposhian and Aposhian, 2006; Zhang et al., 2013b). The imbalance between ROS production and the defensive activity of enzymatic and nonenzymatic antioxidant systems is the root cause of oxidative stress, which is a destructive biological response mechanism for cell growth. MDA is the end product of the peroxidation reaction between ROS and lipids, which can be used as a marker of oxidative stress (Chen et al., 2020; Zhong et al., 2020). In addition, SOD and CAT are the key enzymes of the endogenous antioxidant system and have catalytic inactivation and scavenging effects on ROS. They are also the first line of defence against oxidative damage. GSH, a non-enzymatic antioxidant, playing an effective role in scavenging ROS (Fang et al., 2002). This study found that ATO-induced oxidative damage is embodied as the accumulation of ROS and MDA, the reduction of SOD, CAT and GSH in tissue (Figures 4, 5). This finding is consistent with the results of previous studies (Liang et al., 2020; Zhao et al., 2020). However, HES treatment alleviated ATO-induced oxidative stress by attenuating ROS and MDA levels and stimulating the activity of enzymatic and nonenzymatic antioxidant systems. The result demonstrates that the significant antioxidant effect of HES is an important mechanism for mitigating ATO-induced cardiotoxicity.

Many studies have confirmed that inflammation has played a key role in the pathogenesis of ATO-induced cardiotoxicity (Liang et al., 2020; Zhao et al., 2020). ATO induces the overproduction of ROS, used as signal molecules to activate the intracellular inflammatory cascade and promote the expression of downstream inflammatory cytokines TNF-α and IL-6 (Kang et al., 2019). Liang (Liang et al., 2020) showed that the ATO caused high expression of TNF-α and IL-6 in heart tissue, which was the pathological fundamental factor for cardiac toxicity. Our results confirmed that inflammation was a crucial player in ATO-mediated cardiotoxicity. Thus, a statistically significant increase was noted in TNF-α and IL-6 levels of the ATO group compared to the CONT group. Our results also demonstrated that HES significantly inhibited the increase of ATO-induced pro-inflammatory cytokines and reduced ATO cardiotoxicity dominated by inflammatory responses (Figure 6).

The Bcl-2 family plays a vital role in the process of apoptosis. In addition, Bax is the most representative pro-apoptotic protein in the Bcl-2 family and the main regulator of anti-apoptotic Bcl-2 protein activity. Studies have shown that the mitochondria-mediated endogenous pathway plays a dominant role in ATO-induced apoptosis (Gu et al., 2016; Yun et al., 2016). Cardiomyocytes are rich in mitochondria, and they are the driving force of energy metabolism. A massive amassment of ATO in the mitochondrial matrix causes ROS overproduction, resulting in the activation of Bax and increasing mitochondrial permeability. This induces the activation of caspase cascade reactions and triggers cell apoptosis (Oliveira et al., 2006). Caspase-3 is one of the major executors of apoptosis in the cascade reaction (Adedara et al., 2019; Van Opdenbosch and Lamkanfi, 2019). When cell apoptosis occurs, Caspase-3 is activated through the mitochondrial pathway, which is called cleaved-Caspase-3. However, cleaved-Caspase-3 can degrade DNA replication-related proteins, apoptosis inhibitory proteins and cytoskeleton proteins, etc., thereby promoting apoptosis (Esteban-Fernández de Ávila et al., 2017). We examined the conditions of myocardial mitochondria through TEM. The results showed that ATO caused multiple damages to mitochondria (Figure 7), which were associated with promoting the activation of endogenous apoptotic pathways. Moreover, in Figure 8, western blot results showed that ATO significantly increased the expression levels of Bax, Caspase-3 and cleaved-Caspase-3 and decreased Bcl-2, consistent with the outcomes of previous studies (Liang et al., 2020; Zheng et al., 2021). However, in HES_H_ mice, a significant change is remarked in Bcl-2 expression, conspicuously decreases in Bax, Caspase-3 and cleaved-Caspase-3 expression, and mitochondrial damage was significantly alleviated. The results confirmed the inhibitory effect of HES on myocardial apoptosis.

Oxidative stress, inflammation and apoptosis are involved in ATO-induced cardiotoxicity, of which oxidative stress is a risk factor (Liang et al., 2020; Zheng et al., 2021). Nrf2 plays an indelible role in the regulation of oxidative stress reactions. When Nrf2 is inhibited or inactivated, it aggravates the state of oxidative stress and destroys redox homeostasis. Keap1, an inhibitor of Nrf2 activity, plays a critical role in its regulation. In addition, Keap1 is a “sensor” of oxidative stress (Sihvola and Levonen, 2017). Under oxidative stress, conformational changes in the Keap1 cysteine residues disrupt Keap1-Nrf2 linkage, resulting in Nrf2 nuclear translocation and binding to ARE. This regulates the expression of downstream antioxidant proteins and detoxifies enzymes, thus reducing the damage of cells and tissues through oxidative stress (Canning et al., 2015; Suzuki and Yamamoto, 2015). P62 is a multifunctional stress protein and an important upstream regulator of the Keap1-Nrf2 pathway. Oxidative stress induces up-regulation of p62 protein expression, blocks Keap1-Nrf2 linkage, enhances the interaction of p62 and Keap1 and promotes Nrf2 translocate to the nucleus (Sun et al., 2020). In addition, Nrf2 is the core regulator of downstream antioxidant enzymes and is responsible for the activation of SOD and CAT [ (Jaiswal, 2004; Zhao, 2019)]. In the present study, HES treatment significantly enhanced the expression levels of p62 and Nrf2, inhibited by the ATO (Figure 9). Increased SOD and CAT activities were observed after HES treatment (Figure 5). Thus, HES may activate the Nrf2 pathway in a p62-Keap1 mediated manner, reducing oxidative stress injury and subsequent inflammation and apoptosis.

## Conclusion

**FIGURE 10:**
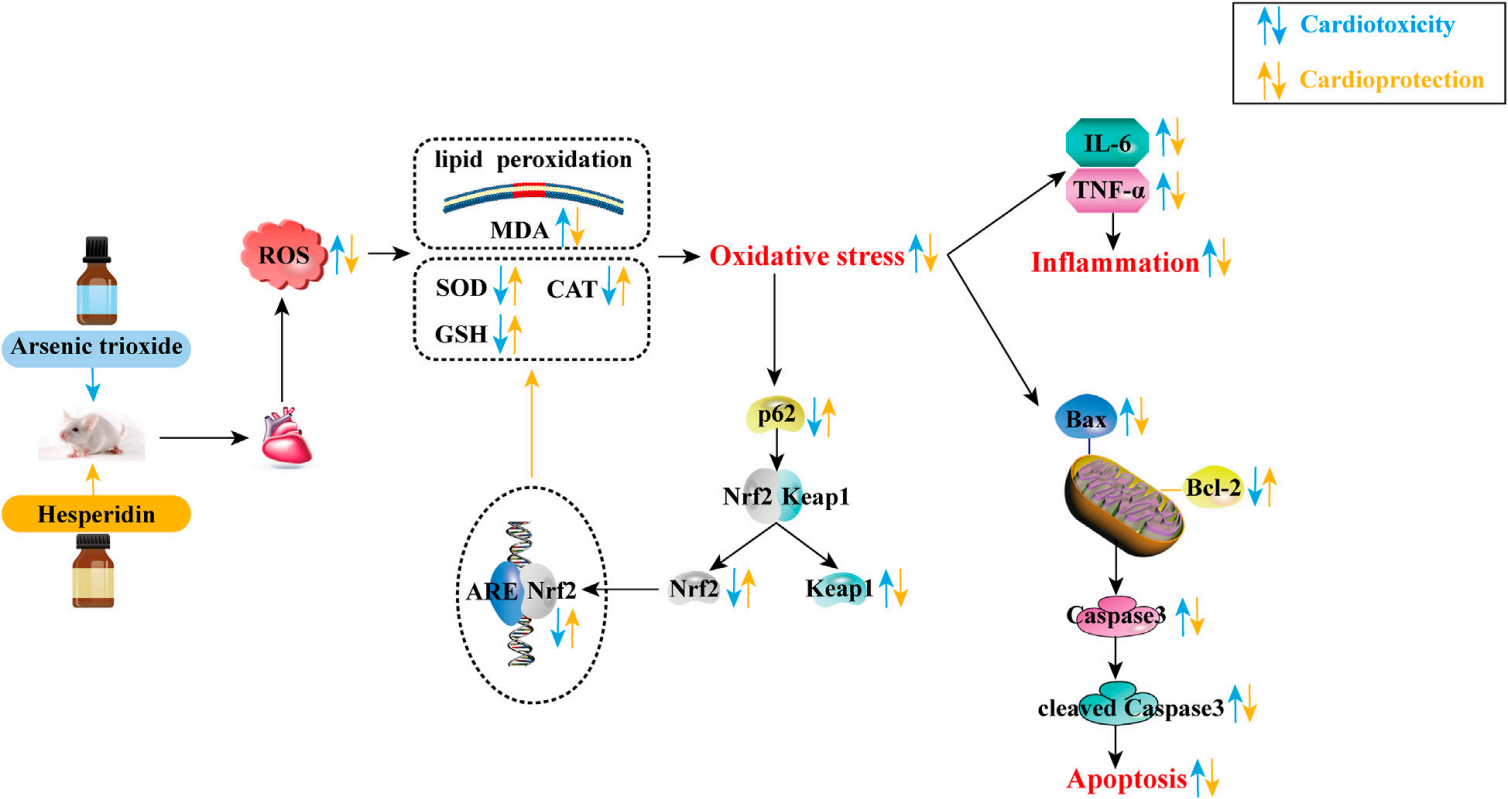
The mechanism for cardioprotection of HES against cardiotoxicity induced by ATO.

This study demonstrates that HES can restrain ATO-induced cardiotoxicity based on the mechanisms of inhibiting oxidative stress, inflammation and apoptosis. The effect involves the activation of the p62-Keap1-Nrf2 signalling pathway (Figure 10). Based on these findings, HES could provide a safe option to resist ATO-induced cardiotoxicity in future anti-cancer clinical therapies.

## Limitations

Mitochondrial damage is assessed using TEM only, and a more comprehensive assessment of mitochondrial damage is required to reveal the protective role of HES against ATO induced cardiotoxicity.

## Data Availability

The original contributions presented in the study are included in the article/supplementary files, further inquiries can be directed to the corresponding authors.
